# Reviewers BJCVS 32.2

**Published:** 2017

The Brazilian Journal of Cardiovascular Surgery (BJCVS) is grateful for the reviewers, listed below, which collaborated in this edition. Without their work would be impossible to keep the high scientific standard of our journal.

Alfredo Inácio Fiorelli
Douglas Bolzan
Gibran Roder Feguri
Gilberto Barbosa
Lindemberg Silveira
Magaly Arrais dos Santos
Marcos Aurélio Barboza de Oliveira
Melchior Lima
Michel Silva Reis
Reinaldo Bestetti
Renata Trimer
Ricardo Lima
Rui M. S. Almeida
Sergio Francisco dos Santos Junior

